# „Resuscitative endovascular balloon occlusion of the aorta“ bei schwer verletzten Patienten im Schockraum: eine Fallserie

**DOI:** 10.1007/s00101-022-01100-3

**Published:** 2022-03-07

**Authors:** Jürgen Knapp, Dominik A. Jakob, Tobias Haltmeier, Beat Lehmann, Wolf E. Hautz

**Affiliations:** 1 Klinik für Anästhesiologie und Schmerztherapie, Inselspital, Universitätsspital Bern, Universität Bern, Bern, Schweiz; 2 Klinik für Anästhesie, Intensivmedizin und Schmerztherapie, Spital Schwyz, Schwyz, Schweiz; 3 Universitäres Notfallzentrum, Inselspital, Universitätsspital Bern, Universität Bern, Bern, Schweiz; 4 Klinik für Viszerale Chirurgie und Medizin, Inselspital, Universitätsspital Bern, Universität Bern, Bern, Schweiz; 5 Klinik für Anästhesiologie und Schmerztherapie, Universitätsspital Bern, Universität Bern, Freiburgstraße, 3010 Bern, Schweiz

**Keywords:** REBOA, Endovaskuläre Therapie, Schwerstverletztenversorgung, Schock, Behandlungsmethoden, REBOA, Endovascular procedures, Multiple trauma, Resuscitation, Shock

## Abstract

Eine Hämorrhagie ist in 30–40 % der Patienten, die im Rahmen eines Traumas versterben, todesursächlich und die häufigste vermeidbare Todesursache. Für nichtkomprimierbare Blutungen im Bereich des Abdomens oder des Beckens wird in den letzten Jahren zunehmend (wieder) die Anwendung der „resuscitative endovascular balloon occlusion of the aorta“ (REBOA) zur temporären Blutungskontrolle diskutiert. Seit August 2020 steht im Schockraum des Universitären Notfallzentrums des Universitätsspital Bern die REBOA als Therapieoption im Rahmen der Schwerverletztenversorgung zur Verfügung. Wir berichten in dieser Fallserie unsere Erfahrungen aus allen 7 Anwendungen im Laufe des ersten Jahres.

## Abstract (en)

Hemorrhage is the cause of death in 30–40% of severely injured patients due to trauma and the most frequent avoidable cause of death. In civilian emergency medical services, the majority of life-threatening hemorrhages are found in incompressible body regions (e.g. abdomen and pelvis). Resuscitative endovascular balloon occlusion of the aorta (REBOA) has therefore been discussed in recent years as a lifesaving procedure for temporary bleeding control in multiple trauma patients. Since August 2020 REBOA is implented in the treatment of seriously injured patients in the emergency department of the University Hospital of Bern. In this case series we report on our experiences in all seven patients in whom we performed this procedure during the first year.

## Einleitung

Bei Schwerverletzen ist die Hämorrhagie verantwortlich für 30–40 % der Todesfälle und gleichzeitig die häufigste potenziell vermeidbare Todesursache [1–5]. Die überwiegende Mehrzahl der lebensbedrohlichen Blutungen in der zivilen Notfallmedizin in Europa findet sich in Becken und Abdomen [1, 6, 7]. Der Einsatz der „resuscitative endovascular balloon occlusion of the aorta“ (REBOA) zur temporären Blutungskontrolle bei diesen Verletzungen hat in den letzten Jahren ein gewisses „Revival“ erfahren. Nachdem die erste Beschreibungen einer aortalen Ballontamponade zur Versorgung einer intraabdominellen Verletzung aus der Militärmedizin zu Zeiten des Koreakrieges stammen [8, 9], fand in den vergangenen Jahrzehnten die Technik im Wesentlichen in einigen gefäßchirurgischen Zentren zur temporären Blutungskontrolle beim rupturiertem Bauchaortenaneurysma Anwendung [10, 11]. Durch die technische Weiterentwicklung des Materials und den Rückgang der verfahrensbedingten Komplikationen wird die REBOA inzwischen wieder v. a. im Rahmen von Beckenfrakturen zur temporären Blutungskontrolle als vielversprechende Methode diskutiert, um die Zeit bis zur operativen oder zur interventionell-angiologischen Blutstillung zu überbrücken [12–15]. In der aktuellen S3-Leitlinie „Polytrauma/Schwerverletztenversorgung“ wird die REBOA im Sinne einer Expertenmeinung (Empfehlungsgrad 0) bei „kreislaufinstabilen Patienten in extremis“ empfohlen [16]. Eine Übersicht über die aktuellen internationalen Leitlinien und leitliniennahen Empfehlungen zum Einsatz der REBOA bietet Tab. 1.

**Table 1:** 

Publikation	Empfehlungen
S3-Leitlinie Polytrauma/Schwerverletztenversorgung (Deutschland) [16]	„Die temporäre endovaskuläre Ballonokklusion der Aorta (REBOA) oder anderer großer Gefäße kann bei kreislaufinstabilen Patienten in extremis zur Wiederherstellung einer zentralen Zirkulation angewandt werden, um das Zeitfenster bis zur definitiven operativen oder interventionellen Therapie zu vergrößern.“ (Empfehlungsgrad 0)
S3-Leitlinie zu Screening, Diagnostik, Therapie und Nachsorge des Bauchaortenaneurysmas (Deutschland) [17]	„Der Einsatz des aortalen Okklusionsballons bei EVAR sollte bei Patienten mit hypovolämischem Schock erwogen werden.“ (Evidenzgrad 4/Empfehlungsgrad B, starker Konsens)
The European guideline on management of major bleeding and coagulopathy following trauma: fifth edition (Europa) [18]	„We suggest that the use of aortic balloon occlusion be considered only under extreme circumstances in patients with pelvic fracture in order to gain time until appropriate bleeding control measures can be implemented.“ (Grade 2C)
Consensus on resuscitative endovascular balloon occlusion of the aorta: A first consensus paper using a Delphi method (internationale Konsenspublikation) [19]	„The expert panel reached consensus that REBOA can be used in austere military setting, emergency departments, operating rooms and intensive care units, but disagrees with the statement that REBOA is feasible in the prehospital setting (20/36, 55.6 %). Panel members reached consensus that REBOA is indicated in the following patient populations: traumatic abdominopelvic hemorrhage, hemorrhage arising from a ruptured aneurysm, patients with severe postpartum hemorrhage (PPH) […]. The physiological parameters to select patients for REBOA use are trauma victims […] with an initial systolic blood pressure of < 90 mm Hg who do not respond at all to initial fluid or blood products and trauma victims with an ATLS class IV hypovolemic shock. Patients in extremis (no pulse, no blood pressure) should not be considered for REBOA with 25/36; 69.4 % of panel members agreeing. […] The panel did not support the statement that trauma victims with a systolic pressure > 90 mm Hg, but with a mechanism of injury suspicious for high early bleeding risk (severe pelvic fracture, positive FAST exam) should be eligible for REBOA (7/36; 19.4 %).“
Joint statement from the American College of Surgeons Committee on Trauma (ACS COT) and the American College of Emergency Physicians (ACEP) regarding the clinical use of Resuscitative Endovascular Balloon Occlusion of the Aorta (REBOA) (USA) [20]	„REBOA is indicated for traumatic life-threatening hemorrhage below the diaphragm in patients in hemorrhagic shock who are unresponsive or transiently responsive to resuscitation.“ „REBOA is indicated for patients arriving in arrest from injury due to presumed life-threatening hemorrhage below the diaphragm. No evidence exists for the recommended duration of arrest and use of REBOA but should be used within the same time period as would resuscitative thoracotomy.“
Resuscitative endovascular balloon occlusion of the aorta in NSW trauma (Australien) [21]	„Currently, there is insufficient high-level evidence on REBOA’s effectiveness for improving mortality outcomes in trauma. […] Uncertainty around which patient cohort is likely to most benefit (i.e. survival benefit) from REBOA remains a fundamental question; identifying the optimal patient group, where and when REBOA is implemented are some crucial questions, when answered, may provide greater clarity. TIC recommends further prospective studies are necessary to understand the role of REBOA in torso haemorrhage in trauma and which patient group is likely to benefit from REBOA intervention.“
Clinical use of resuscitative endovascular balloon occlusion of the aorta (REBOA) in civilian trauma systems in the USA, 2019: a joint statement from the American College of Surgeons Committee on Trauma, the American College of Emergency Physicians, the National Association of Emergency Medical Services Physicians and the National Association of Emergency Medical Technicians (USA) [22]	„There is no high-grade evidence demonstrating that REBOA improves outcomes or survival compared with standard treatment of severe traumatic hemorrhage. […] At a small number of high-volume trauma centers experienced with this procedure, REBOA has emerged as a protocolized option in select patients with non-compressible torso trauma. […] REBOA is a tool that should only be employed as part of a larger system of damage control resuscitation, definitive hemorrhage control, and postoperative critical care. It is used to temporize patients at high risk of mortality from non-compressible torso hemorrhage.“
Resuscitative endovascular balloon occlusion of the aorta (REBOA) in patients with major trauma and uncontrolled haemorrhagic shock: a systematic review with meta-analysis (Italien, Metaanalyse im Rahmen der Leitlinien-Entwicklung zur Schwerverletztenversorgung für das Istituto Superiore di Sanità) [23]	„Our findings on overall mortality suggest a positive effect of REBOA among non-compressible torso injuries when compared to RT but no differences compared to no-REBOA. Variability in indications and patient characteristics prevents any conclusion deserving further investigation. REBOA should be promoted in specific training programs in an experimental setting in order to test its effectiveness and a randomized trial should be planned.“

*ATLS* advanced trauma life support, *EVAR* endovascular aortic repair, *FAST* focused assessment with sonography in trauma, *NSW* New South Wales, *TIC* Trauma Innovation Committee

**Figure 1:**
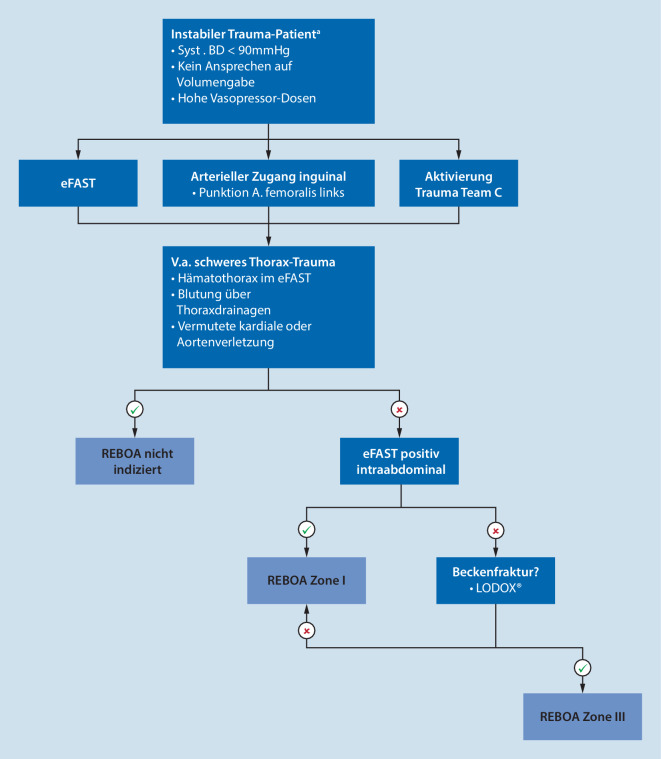

Wir haben im Universitären Notfallzentrum des Universitätsspitals Bern seit 2020 einen Algorithmus zur Durchführung einer REBOA bei hämodynamisch instabilen Traumapatienten etabliert (Abb. 1). Seither wurde das REBOA-Manöver über den Zeitraum von 12 Monaten bei 7 Patienten durchgeführt. Im Folgenden möchten wir konsekutiv alle Fälle kurz berichten und unsere Erfahrungen zur Diskussion stellen. Die in den einzelnen Fallberichten geschilderten Verletzungen sind die Diagnosen, die nach Abschluss der kompletten Diagnostik (inklusive Computertomographie) im Rahmen der Schockraumversorgung gestellt wurden. Die Entscheidung zur REBOA musste in den meisten Fällen aber sehr frühzeitig und meist nur anhand der Informationen zur Unfallkinematik, der Vitalparameter und der Erstuntersuchung (in der Regel inklusive fokussierter Sonographie [„extended focused assessment with sonography for trauma“, eFAST] und evtl. Röntgenaufnahme des Thorax und Beckens) im Schockraum gestellt werden.

## Fallberichte

### Fall 1

#### Unfallmechanismus.

Der Patient wurde bei Baumfällarbeiten zwischen 2 Bäumen eingeklemmt.

#### Initiale Arbeitsdiagnose.

Beckentrauma, stumpfes Thorax- und Abdominaltrauma.

#### Hämodynamische Situation.

Bei Aufnahme in den Schockraum hämodynamisch instabil. Mittlerer arterieller Druck (MAD) 50–60 mm Hg unter Noradrenalin bis 0,1 µg/kgKG und min.

#### REBOA-Manöver.

Problemlose, ultraschallgestützte Einlage einer 7‑F-Schleuse femoral-arteriell rechts und konsekutives Einbringen einer ER-REBOA® (Fa. Prytime Medical, Texas, USA) in Zone III innerhalb von 9 min (gemessen von der Entscheidung für das REBOA-Manöver bis zur Ballonokklusion der Aorta). Lagekontrolle durch Ableiten arterieller Druckkurven an Schleuse und Katheterspitze. Nach REBOA MAD 70–80 mm Hg, kein Noradrenalinbedarf mehr.

Umstellung auf partielle REBOA (d. h. nur noch partiell geblockter Ballon, sodass in der invasiv gemessenen Blutdruckkurve distal der Aortenokklusion wieder eine leichte Pulsation zu erkennen ist) und vollständiger Rückzug des Katheters innerhalb der folgenden 45 min unter massiver Volumensubstitution (Kristalloide, Erythrozytenkonzentrate und Frischplasma) und nach Stabilisierung und Anlegen einer Beckenzwinge schließlich Verlegung in die Angiographie-Suite. Die einliegende femoral-arterielle Schleuse konnte für die Embolisation der pelvinen Blutungsquelle genutzt werden.

#### Wesentliche Verletzungen.

Beckenfraktur (C-Verletzung) mit bilateraler Sakrumfraktur sowie Schambeinastfraktur rechts und Sprengung des Iliosakralgelenks, Rippenserienfraktur rechts mit Pneumothorax, große Morel-Lavallée-Verletzung gluteal rechtsseitig, Milzlazeration I°.

#### Behandlungsergebnis.

Exitus letalis 22 Tage nach dem Trauma nach Entscheid für eine palliative Therapie bei septischem Multiorganversagen bei im Verlauf diagnostizierter Rektum- und Dünndarmperforation im Rahmen eines Abrisses des Mesenteriums sowie multiplen ischämischen Grenzzoneninfarkten im Mediastromgebiet bei zerebrovaskulärer Verschlusskrankheit.

### Fall 2

#### Unfallmechanismus.

Sturz aus dem 3. Stock.

#### Initiale Arbeitsdiagnose.

Schweres Polytrauma mit führendem Schädel-Hirn-Trauma, eFAST: wenige freie Flüssigkeit im Morison-Pouch.

#### Hämodynamische Situation.

Hämodynamisch instabil, MAD 30–45 mm Hg unter 0,08 µg/kgKG und min Noradrenalin, Herzfrequenz (HF) 130/min.

#### REBOA-Manöver.

Problemlose, ultraschallgestützte Einlage einer 7‑F-Schleuse femoral-arteriell rechts und konsekutives Einbringen einer ER-REBOA innerhalb von 13 min. Entgegen den klinikinternen Vorgaben (Abb. 1) wird der Ballon trotz positivem eFAST in Zone III, nicht in Zone I platziert. Lagekontrolle durch Ableiten arterieller Druckkurven an Schleuse und Katheterspitze. Umstellung auf partielle REBOA unter kontinuierlicher Volumengabe mit kristalloiden Infusionslösungen und Blutprodukten und vollständige Entfernung des Katheters nach 90 min.

Nach REBOA: MAD 80–100 mm Hg unter 0,02–0,04 µg/kgKG und min Noradrenalin, HF 100–110/min.

#### Wesentliche Verletzungen

Traumatische Subarachnoidalblutung, Dissektion der A. vertebralis links im V_2_-Segment, instabile Wirbelkörperfraktur Halswirbelkörper (HWK) 3/4, Lungenlazeration links mit kleinem Pneumothorax.

#### Behandlungsergebnis.

Exitus letalis 33 Tage nach dem Trauma nach Entscheid für eine palliative Therapie bei fehlender Aufwachreaktion nach Ausschluss reversibler Ursachen und irreversibler hoher Schädigung des Rückenmarks in Höhe HWK 3/4.

### Fall 3

#### Unfallmechanismus.

Patient wurde von einem Zug erfasst.

#### Initiale Arbeitsdiagnose.

Beckentrauma, Thoraxtrauma, beidseitige Unterschenkelamputation, eFAST: Pneumothorax links, keine freie Flüssigkeit im Abdomen.

#### Hämodynamische Situation.

Hämodynamisch instabil, MAD 70 mm Hg unter 0,25 µg/kgKG und min Noradrenalin, Herzfrequenz 140/min.

#### REBOA-Manöver.

Beim wachen Patienten frustrane Schleuseneinlage unter Ultraschallkontrolle in der rechten Femoralarterie (Draht nicht zu positionieren). Schließlich problemlose, ultraschallgestützte Einlage einer 7‑F-Schleuse femoral-arteriell links und konsekutives Einbringen eines ER-REBOA®-Katheters innerhalb von 7 min in Zone III. Aortenokklusion zeitgleich mit Einleitung der Intubationsnarkose. Lagekontrolle durch Ableiten arterieller Druckkurven an Schleuse und Katheterspitze. Umstellung auf partielle REBOA unter kontinuierlicher Volumengabe und Übernahme des Patienten mit partiell geblocktem Katheter in den OP.

Nach REBOA: MAD 80–100 mm Hg ohne Katecholamine, HF 80–100/min.

#### Wesentliche Verletzungen.

Rippenserienfraktur links, kleiner Hämatopneumothorax links, Sternumfraktur, Acetabulumfraktur rechts, beidseitige Hüftluxation, beidseitige komplette Unterschenkelamputation, blutend trotz straff anliegender Tourniquets.

#### Behandlungsergebnis.

Exitus letalis 18 Tage nach dem Trauma nach Entscheid zur palliativen Therapie bei malignem Mediainfarkt unklarer Ursache.

### Fall 4

#### Unfallmechanismus.

Fahrradfahrer von Lkw überrollt.

#### Initiale Arbeitsdiagnose.

Beckenfraktur, ausgedehnte Weichteilverletzungen, eFAST: negativ.

#### Hämodynamische Situation.

Hämodynamisch instabil, MAD 70–80 mm Hg unter Noradrenalin auf 0,02–0,05 µg/kgKG und min und Massivtransfusion, HF 90–130/min.

#### REBOA-Manöver.

Bei initial mäßiger Volumenresponse Einlage einer 7‑F-Schleuse femoral-arteriell links unter Sicht (aufgrund der ausgedehnten Décollement-Verletzung) und beim wachen Patienten problemlos. Anlage einer Beckenzwinge im Schockraum nach Intubationsnarkose. Im Verlauf bei zunehmender hämodynamischer Instabilität Einbringen eines ER-REBOA®-Katheters über die Schleuse in Zone II (Ziel war Zone III) und Verlegung des Patienten in den OP.

Nach REBOA: MAD 60–80 mm Hg ohne Katecholamine, HF 60–70/min.

#### Wesentliche Verletzungen.

Décollement von 35–40 % der Körperoberfläche an Rumpf und Oberschenkel beidseits, Beckenfraktur (C-Verletzung) mit spinopelviner Dissoziation, Trümmerfraktur des Sakrums, bilaterale Schambeinasttrümmerfraktur, Impressionsfraktur des rechten Acetabulums mit zentraler Luxation des Hüftkopfes, Dünndarmperforation, Harnblasenruptur.

#### Behandlungsergebnis.

Verlegung in ein Verbrennungszentrum aufgrund der ausgedehnten Weichteilverletzungen am 13. Tag nach dem Trauma, wach, orientiert, unter suffizienter Analgesie.

### Fall 5

#### Unfallmechanismus.

Motorradunfall.

#### Initiale Arbeitsdiagnose.

Thoraxtrauma, Abdominaltrauma, Beckentrauma, eFAST: Hämatopneumothorax rechts, freie Flüssigkeit im Abdomen.

#### Hämodynamische Situation.

Hämodynamisch instabil, MAD 60–70 mm Hg unter Noradrenalin 0,07 µg/kgKG und min, HF 100–110/min.

#### REBOA-Manöver.

Bei Eröffnung des Beckengurts in Vorbereitung zur operativen Stabilisierung nach Abschluss der Diagnostik kam es zur massiven hämodynamischen Instabilität, sodass wir uns zur sofortigen Anlage eines REBOA-Katheters entschieden. Einlage der Schleuse problemlos unter Ultraschallkontrolle und Positionierung des Ballons in Zone I.

Nach REBOA: MAD 60–70 mm Hg unter rückläufiger Noradrenalindosierung bis auf 0,01 µg/kgKG und min, HF 70–80/min. Übernahme in den OP zur operativen Stabilisierung des Beckens.

#### Wesentliche Verletzungen.

Contusio cordis, kleiner Hämatopneumothorax rechts, dislozierte Rippenserienfraktur rechts, Milzlazeration und Leberlazeration II°, aktive intraabdominale Blutung bei Lazeration A. hepatica und Mesoeinriss, komplexe Beckenfraktur (C-Verletzung) mit aktiver Blutung, Blasenruptur.

#### Behandlungsergebnis.

Entlassung am 68. Tag nach dem Trauma in die Rehabilitation in gutem Allgemeinzustand.

### Fall 6

#### Unfallmechanismus.

Der Patient wurde von einem Lkw überrollt. Kurz nach Eintreffen des Rettungsdienstes wird der Patient reanimationspflichtig bei pulsloser elektrischer Aktivität des Herzens (PEA).

#### Initiale Arbeitsdiagnose.

Traumatischer Herz-Kreislauf-Stillstand.

#### Hämodynamische Situation und REBOA-Manöver.

Bei Aufnahme in den Schockraum war der Patient nach wie vor reanimationspflichtig. Daher entschlossen wir uns zur sofortigen linksseitigen Thorakotomie und Klemmung der Aorta (Zeit von Ankunft im Schockraum bis Klemmen der Aorta 4 min), parallel wurde rechtsseitig eine Thoraxdrainage in Bülau-Position eingelegt. Unter zusätzlicher Massivtransfusion konnte so innerhalb von 5 min eine Wiederherstellung eines Spontankreislaufs („restoration of spontaneous circulation“, ROSC) erzielt werden. Im anschließend durchgeführten eFAST fand sich abdominell keine freie Flüssigkeit, sodass während der Vorbereitungen zur Anlage einer Beckenzwinge eine femoral-arterielle Schleuse (mittels „Cut-down“-Technik) und hierüber eine REBOA in Zone III platziert wurden. Dieses Manöver konnte innerhalb von 5 min und ohne erneute hämodynamische Instabilität durchgeführt werden.

#### Wesentliche Verletzungen.

Die Aufnahme in den Schockraum erfolgt unter kardiopulmonaler Reanimation bei pulsloser elektrischer Aktivität des Herzens (PEA). „Open book“-Verletzung des Beckens, Rippenserienfrakturen beidseits.

#### Behandlungsergebnis.

Im Laufe der operativen Versorgung des Patienten kam es trotz Massivtransfusion und intensiver Substitution von Gerinnungsfaktoren zur diffusen Blutungstendenz in Abdomen und Thorax, die auch chirurgisch nicht weiter zu kontrollieren war. Daher wurde interdisziplinär die Entscheidung zum Therapieabbruch getroffen. Exitus letalis 5 h nach Aufnahme.

### Fall 7

#### Unfallmechanismus.

Gleitschirmabsturz aus ca. 30 m Höhe. Kurz nach Eintreffen des Rettungshubschraubers wird der Patient während der Versorgung reanimationspflichtig, ROSC prähospital nach einer Reanimationsdauer von 20 min.

#### Initiale Arbeitsdiagnose.

Traumatischer Herz-Kreislauf-Stillstand, eFAST: freie Flüssigkeit im Koller-Pouch, wenig Perikarderguss, hämodynamisch nicht relevant.

#### Hämodynamische Situation.

Zwei Minuten nach Aufnahme des Patienten in den Schockraum kommt es erneut zu einem Kreislaufstillstand bei PEA.

#### REBOA-Manöver.

Problemlose, ultraschallgesteuerte Einlage einer 7‑F-Schleuse femoral-arteriell rechts. Unter nun notwendiger kardiopulmonaler Reanimation wird die bereits vor Eintritt des erneuten Herz-Kreislauf-Stillstandes begonnene REBOA-Einlage nun innerhalb von 2 min durch Einlage eines ER-REBOA®-Katheters in Zone I beendet. Insgesamt dauerte die REBOA-Anlage 7 min. Unmittelbar nach Okklusion der Aorta ROSC. Rückzug in Zone III nach Abschluss der Schockraumdiagnostik, da die Computertomographie keine relevante intraabdominelle Flüssigkeit zeigte.

#### Wesentliche Verletzungen.

Prähospitaler traumatischer Herz-Kreislauf-Stillstand mit primär erfolgreichem ROSC, schweres Schädel-Hirn-Trauma mit Subarachnoidalblutung, stumpfes Abdominaltrauma mit freier Flüssigkeit perisplenisch, multiple instabile Wirbelkörperfrakturen (HWK 5, Brustwirbelkörper 3, 4 und 11, Lendenwirbelkörper 1, 4 und 5), dislozierte offene Femurfraktur links.

#### Behandlungsergebnis.

Die computertomographische Diagnostik zeigte bereits ein schweres diffuses Hirnödem, schon mit Zeichen der hypoxischen Hirnschädigung, Kornealreflexe ließen sich nicht auslösen (nach Ausschluss einer neuromuskulären Blockade), beide Pupillen waren weit und lichtstarr. Daher entschlossen wir uns noch im Schockraum zur Einleitung einer palliativen Therapie. Der Patient verstarb 5 h nach Aufnahme auf der Intensivstation im Beisein der Familie.

Eine Übersicht über die geschilderten Fälle bietet Tab. 2.

**Table 2:** 

Fall, Geschlecht, Alter in Jahren	Führende Verletzung	Zeit bis Abschluss des eFAST	Zeit im Schockraum bis REBOA	Zeit bis Abschluss CT-Diagnostik	Gesamtzeit der Schockraumversorgung	24-h-Überleben	Krankenhausentlassung	Todesursache
Fall 1, m, 68	Schweres Beckentrauma	3 min	32 min	50 min	160 min (inkl. Beckenzwinge)	Ja	Nein	Septisches Multiorganversagen
Fall 2, w, 58	Schweres Schädel-Hirn-Trauma, Wirbelsäulentrauma	15 min	15 min	67 min	225 min (inkl. externe Ventrikeldrainage)	Ja	Nein	Schweres Schädel-Hirn-Trauma
Fall 3, m, 33	Schweres Beckentrauma, beidseitige Unterschenkelamputation	6 min	21 min	45 min	126 min	Ja	Nein	Maligner Mediainfarkt
Fall 4, m, 40	Schweres Beckentrauma	2 min	40 min	78 min	176 min (inkl. Beckenzwinge)	Ja	Ja	–
Fall 5, m, 62	Schweres Abdominal- und Beckentrauma	5 min	–^a^	76 min	262 min (Wartezeit auf OP)	Ja	Ja	–
Fall 6, m, 64	Traumatischer Herz-Kreislauf-Stillstand	13 min	Sofortige linksseitige Thorakotomie, Klemmung der Aorta thoracica und Bülau-Drainage rechtsseitig, Umstellung auf REBOA nach 12 min	94 min	99 min	Nein	–	Nichtbeherrschbare abdominelle und thorakale Blutung nach traumatischem Herz-Kreislauf-Stillstand
Fall 7, m, 76	Traumatischer Herz-Kreislauf-Stillstand	3 min	7 min	36 min	98 min (Überleitung in palliative Therapie)	Nein	–	Schweres diffuses Hirnödem nach prähospitalem traumatischem Herz-Kreislauf-Stillstand bei Polytrauma

*m* männlich, *w* weiblich, *eFAST* „extended focused assessment with sonography for trauma“, *CT* Computertomographie, *REBOA* resuscitative endovascular balloon occlusion of the aorta

^a^Die Entscheidung zur REBOA fiel in diesem Fall erst, als es nach Abschluss der Schockraumdiagnostik bei der Eröffnung des Beckengurts zur Vorbereitung für die operative Versorgung zur hämodynamischen Instabilität kam

## Diskussion

Die Zahl der Patienten, bei denen wir uns für die Durchführung eines REBOA-Manövers entschieden, überstieg mit 7 innerhalb eines Jahres deutlich unsere Erwartungen. Diese Zahl lag auch deutlich über den Abschätzungen aus dem Traumaregister der Deutschen Gesellschaft für Unfallchirurgie (DGU). Diese ergaben, dass in einem überregionalen Traumazentrum mindestens ein Patient/Jahr zu erwarten ist, bei dem eine REBOA indiziert ist [24]. Da das Verfahren momentan noch nur von einer kleinen Gruppe an Ärzten angewandt wird, stand die Option zur REBOA nicht täglich und nicht rund um die Uhr zur Verfügung (in der Regel nur tagsüber und werktags). Daher liegt die tatsächliche Zahl der Patienten, bei denen eine REBOA indiziert gewesen wäre, vermutlich noch höher.

Die geschilderten Fälle zeigen, dass die initiale hämodynamische Stabilisierung mittels REBOA gut gelingt. Nur einer der Patienten verstarb (nach Entscheid für eine palliative Therapie) infolge einer auch chirurgisch nicht weiter kontrollierbaren Hämorrhagie nach sowohl prähospitaler als auch innerklinisch notwendiger mechanischer Reanimation (vor REBOA). Schwerwiegende Komplikationen durch die REBOA konnten wir bei den 7 Patienten nicht beobachten. In einem Fall wurde der Ballon mutmaßlich beim Einführen durch die Schleuse beschädigt, und ein Aortenverschluss war nicht mehr möglich. Mit einem neuen REBOA-Katheter gelang das Manöver dann im zweiten Versuch. Bei einem Patienten konnte computertomographisch eine Dissektion der Arterienwand im Bereich der A. femoralis nachgewiesen werden, die aber differenzialdiagnostisch auch traumatisch bedingt gewesen sein könnte. Klinisch blieb diese Dissektion für den Patienten folgenlos.

Hinsichtlich der Indikationsstellung kann festgestellt werden, dass, retrospektiv betrachtet, eine REBOA bei einem Patienten (Fall 2) nicht indiziert war. Die Unfallkinematik und die hämodynamische Instabilität bei Aufnahme haben uns in diesem Fall zur Durchführung des REBOA-Manövers veranlasst, wodurch auch eine Stabilisierung des Patienten gelang. Allerdings zeigte sich in der computertomographischen Diagnostik keine Verletzung im Bereich des Beckens oder Abdomens. Die ausgeprägte Hypotonie könnte durch einen neurogenen Schock bei hoher Rückenmarkverletzung verursacht gewesen sein, das gute hämodynamische Ansprechen auf die REBOA durch die massive Erhöhung der Nachlast.

Bei reanimationspflichtigen Patienten mit möglichen thorakalen Verletzungen ist die Thorakotomie („resuscitative thoracotomy“) der REBOA vorzuziehen [13]. Daher entschlossen wir uns im Fall 6 primär zur linksseitigen Thorakotomie mit Klemmung der Aorta. Nachdem hierunter rasch ein ROSC erzielt werden konnte, die FAST-Untersuchung abdominell keine freie Flüssigkeit zeigte, wechselten wir bei klinisch sicherer Beckenfraktur auf eine REBOA in der Zone III, um die thorakale Aortenklemme entfernen zu können und dadurch eine länger dauernde Ischämie der abdominellen Organe zu vermeiden.

Unsere Erfahrung aus dieser Fallserie zeigt, dass das REBOA-Manöver an sich meist innerhalb von wenigen Minuten gelingt. In einigen Fällen ging jedoch mit der Entscheidungsfindung zu diesem Manöver wertvolle Zeit verloren. Dies ist sicher z. T. darauf zurückzuführen, dass die REBOA im Rahmen der Schockraumbehandlung in unserem Notfallzentrum noch kein breit etabliertes Verfahren ist, sodass Unsicherheiten bezüglich der korrekten Indikation und der – insbesondere im Hinblick auf den Zeitbedarf – sinnvollen Durchführbarkeit des Manövers zu Verzögerungen führten. Mit zunehmender Erfahrung und Standardisierung der Anwendung könnten die Entscheidungsprozesse zukünftig schneller ablaufen. Insbesondere die Etablierung von „standard operating procedures“ (SOP), gemeinsames Teamtraining und ein gutes Briefing bei der Vorbereitung auf die Schockraumversorgung eines schwer verletzten Patienten könnten zukünftig die Zeit bis zur Durchführung einer REBOA verkürzen. Unserer Erfahrung nach sind ein Debriefing unmittelbar nach Abschluss der Schockraumversorgung und die postakute Aufarbeitung und kritische Diskussion der Fälle beispielsweise im Rahmen eines Trauma-Board, wie dies am Inselspital Bern monatlich erfolgt, sehr hilfreich.

Als weitere Möglichkeit, den Zeitbedarf bis zum Abschluss des REBOA-Manövers zu reduzieren, diskutieren wir aktuell die standardmäßige Anlage der arteriellen Kanüle zur invasiven Blutdruckmessung in die A. femoralis bei hämodynamisch kompromittierten Patienten mit vom Unfallmechanismus her möglicher Indikation für den Einsatz der REBOA. So kann dieser Zugang jederzeit zum Einbringen der Schleuse genutzt werden, wenn die Entscheidung zur REBOA getroffen ist. Diese Idee zur Optimierung des zeitgerechten Einsatzes wird auch durch ein internationales Konsensus-Statement zur REBOA mit einer Zustimmung von 70 % (28 von 40 Experten) unterstützt [19].

Essenziell bei der Einführung eines solchen Verfahrens ist auch die gute Absprache zwischen den durchführenden Fachabteilungen (in der Regel Notfallmedizin und/oder Anästhesie) und den weiteren an der Schockraumversorgung beteiligten Disziplinen, insbesondere den operativen Fächern. Im angloamerikanischen Raum wird die durch Ballonokklusion der Aorta gewonnene Zeit üblicherweise für eine unmittelbare notfallmäßige Laparotomie genutzt [25]. In allen oben beschriebenen Fällen an unserem Zentrum haben sich die beteiligten operativen Kollegen gegen eine unmittelbare Laparotomie und stattdessen für eine weiterführende Diagnostik durch Computertomographie entschieden. Bei allen Patienten konnte nach REBOA trotz initial extremer hämodynamischer Instabilität die notwendige computertomografische Diagnostik unter deutlich stabileren Bedingungen komplikationslos durchgeführt werden. In Fall 5 wurde die REBOA erst nach Abschluss der Computertomographie durchgeführt, nachdem der Patient beim Öffnen des Beckengurts instabil wurde. Die Narkoseeinleitung und weitere Vorbereitung zur operativen Versorgung verliefen darunter problemlos. Essenziell ist in diesem Zusammenhang aber festzuhalten, dass es sich bei der REBOA lediglich um eine temporäre Maßnahme zur Blutungskotrolle handelt und die weitere Versorgung (Bildgebung, Vorbereitung zur Operation oder angiologischen Intervention) unverzüglich erfolgt.

Ein paar weitere Fragen, die in Diskussionen zum Einsatz der REBOA immer wieder auftreten, können wir im Rückblick auf das eine Jahr der Anwendung in unserem Notfallzentrum beantworten:

### REBOA und bildgebende Diagnostik bei Polytrauma

Hinsichtlich der computertomografischen Diagnostik muss insbesondere geklärt werden, wie mit der REBOA während der arteriellen Phase der „Traumaspirale“ verfahren wird, da durch den Aortenverschluss relevante Blutungen distal davon möglicherweise nicht mehr sicher diagnostiziert werden können. Bei kompletter Deflation des Ballons für die arterielle Phase muss wieder mit schwerer hämodynamischer Instabilität gerechnet werden. Möglich ist hier eine partielle Deflation („partial REBOA“, p‑REBOA). Hier besteht aber die Gefahr, dass der Ballon während der partiellen Deflation disloziert. Wir fahren daher inzwischen zunächst die arterielle Phase einer Traumaspirale mit komplett geblocktem Ballon und wiederholen diese evtl. mit nur teilweise geblocktem Ballon nochmals, falls relevante Hämatome oder unklare Extravasate von Kontrastmittel distal des Ballons festgestellt werden und es die hämodynamische Situation erlaubt. Eine gute Übersicht zu dieser Problematik bietet [26].

### Füllungsvolumen des Ballons

Eine weitere oft diskutierte Frage insbesondere vor dem Hintergrund des variablen Durchmessers der Aorta und der Gefahr einer Verletzung der Aortenwand bei zu starker Füllung des Ballons ist die Steuerung des Füllungsvolumens bei Anwendung der REBOA. Bei gefäßchirurgischen Eingriffen wird das korrekte Füllungsvolumen fluoroskopisch kontrolliert. Dies ist im Schockraum unmöglich. Eine Alternative zur Steuerung des Ballonvolumens ist das Ableiten des invasiv gemessen Blutdrucks an der Schleuse und ein Füllen des Ballons bis zum Verschwinden der pulsatilen Blutdruckwelle. Auch diese Variante erweist sich im Geschehen eines Schockraums häufig als nicht praktikabel und zu zeitaufwändig. Daher verwenden wir für den bei uns verwendeten ER-REBOA®-Katheter inzwischen einen kleinen Einwegdruckmesser (COMPASS®, Fa. Centurion Medical Products, Michigan, USA), der in Reihe zwischen der Spritze und dem Konnektor zum Ballon des REBOA-Katheters geschaltet wird, und schlagen eine initiale Füllung des Ballons auf einen Druck von 160 mm Hg vor [27].

### Materialvorhaltung

Eine aktuelle Ist-Analyse zum Schockraummanagement in Deutschland ergab, dass nur 12 % der Kliniken der Maximalversorgung und sogar nur 3 % der Kliniken der Basisversorgung in Deutschland das Material für eine REBOA vorhalten [28]. Dies ist angesichts der Tatsache, dass die REBOA kein breit etabliertes Verfahren ist, verständlich. Für große (überregionale) Traumazentren, die regelmäßig Patienten mit schweren Beckenverletzungen versorgen, könnten eine Etablierung des Verfahrens und entsprechende Materialvorhaltung aber sinnvoll sein. Ob eine Vorhaltung in kleineren Kliniken sinnvoll ist, um entsprechende Patienten für eine Sekundärverlegung in ein überregionales Traumazentrum zu stabilisieren, lässt sich aus der aktuell vorliegenden Literatur nicht beantworten.

## Fazit für die Praxis

Durch REBOA konnte bei allen 7 Patienten eine hämodynamische Stabilisierung erreicht werden, die eine computertomografische Diagnostik und ggf. Operationsvorbereitung unter relativ stabilen Bedingungen ermöglichte. Insbesondere bei der initialen Stabilisierung von hämodynamisch instabilen Patienten mit schwerem Beckentrauma sahen wir klinisch einen guten Nutzen. Schwerwiegende Komplikationen durch dieses Verfahren wurden in unserer Fallserie nicht beobachtet. Optimierungsbedarf sehen wir in der Indikationsstellung (insbesondere der Zeit bis zur Entscheidungsfindung) sowie der Ausbildung und der Aufrechterhaltung des Trainings in der Breite des Teams.
